# Optimization of anti-ADAMTS13 antibodies for the treatment of ADAMTS13-related bleeding disorder in patients receiving circulatory assist device support

**DOI:** 10.1038/s41598-021-01696-3

**Published:** 2021-11-16

**Authors:** Toshihiro Ito, Takeharu Minamitani, Masaki Hayakawa, Ryota Otsubo, Hiroki Akiba, Kouhei Tsumoto, Masanori Matsumoto, Teruhito Yasui

**Affiliations:** 1 Laboratory of Proteome Research, National Institutes of Biomedical Innovation, Health and Nutrition (NIBIOHN), 7-6-8 Saito-Asagi, Ibaraki City, Osaka 567-0085 Japan; 2 Laboratory of Infectious Diseases and Immunity, National Institutes of Biomedical Innovation, Health and Nutrition (NIBIOHN), 7-6-8 Saito-Asagi, Ibaraki City, Osaka 567-0085 Japan; 3 Laboratory of Immunobiologics Evaluation, Center for Vaccine and Adjuvant Research (CVAR), National Institutes of Biomedical Innovation, Health and Nutrition (NIBIOHN), 7-6-8 Saito-Asagi, Ibaraki City, Osaka 567-0085 Japan; 4 Department of Blood Transfusion Medicine, Nara Medical University, 840 Shijo-cho, Kashihara City, Nara 634-8522 Japan; 5 Laboratory of Advanced Biopharmaceuticals, Center for Drug Design Research (CDDR), National Institutes of Biomedical Innovation, Health and Nutrition (NIBIOHN), 7-6-8 Saito-Asagi, Ibaraki City, Osaka 567-0085 Japan; 6 Graduate School of Pharmaceutical Sciences, Kyoto University, 46-29 Yoshida-shimoadachicho, Sakyo-ku, Kyoto, 606-8501 Japan; 7 Center for Drug Design Research (CDDR), National Institutes of Biomedical Innovation, Health and Nutrition (NIBIOHN), 7-6-8 Saito-Asagi, Ibaraki City, Osaka 567-0085 Japan; 8 Medical Proteomics Laboratory, The Institute of Medical Science, The University of Tokyo, 4-6-1 Shirokanedai, Minato-ku, Tokyo, 108-8639 Japan; 9 Department of Bioengineering, School of Engineering, The University of Tokyo, 7-3-1 Hongo, Bunkyo-Ku, Tokyo, 113-8656 Japan; 10 Laboratory of Pharmaceutical Integrated Omics, Department of Pharmaceutical Engineering, Facility of Engineering, Toyama Prefectural University, 5180 Kurokawa, Imizu, Toyama 939-0398 Japan; 11 Toyama Prefectural Institute for Pharmaceutical Research, Imizu-City, 17-1 Nakataikoyama, Toyama, 939-0363 Japan

**Keywords:** Biochemistry, Computational biology and bioinformatics, Medical research, Molecular medicine

## Abstract

ADAMTS13 (a disintegrin-like and metalloproteinase with thrombospondin type-1 motif 13)-related bleeding disorder has been frequently observed as a life-threatening clinical complication in patients carrying a circulatory assist device. Currently, treatment modalities for the bleeding disorder are very limited and not always successful. To address the unmet medical need, we constructed humanized antibodies of mouse anti-ADAMTS13 antibody A10 (mA10) by using complementarity-determining region (CDR) grafting techniques with human antibody frameworks, 8A7 and 16E8. The characteristics of the two humanized A10 antibodies, namely A10/8A7 and A10/16E8, were assessed in vitro and in silico. Among the two humanized A10 antibodies, the binding affinity of A10/16E8 to ADAMTS13 was comparable to that of mA10 and human-mouse chimeric A10. In addition, A10/16E8 largely inhibited the ADAMTS13 activity in vitro. The results indicated that A10/16E8 retained the binding affinity and inhibitory activity of mA10. To compare the antibody structures, we performed antibody structure modeling and structural similarity analysis in silico. As a result, A10/16E8 showed higher structural similarity to mA10, compared with A10/8A7, suggesting that A10/16E8 retains a native structure of mA10 as well as its antigen binding affinity and activity. A10/16E8 has great potential as a therapeutic agent for ADAMTS13-related bleeding disorder.

## Introduction

ADAMTS13 (a disintegrin-like and metalloproteinase with thrombospondin type-1 motif 13), a member of the ADAMTS family, is a multi-domain metalloproteinase composed of a signal peptide, a propeptide, a metalloprotease domain, a disintegrin domain, a thrombospondin-1 (TSP1) domain, a cysteine-rich domain, spacer domain, additional seven TSP1 repeats, and two complement component C1r/C1s, Uegf, and Bmp1 (CUB) domains^1^. ADAMTS13 specifically cleaves the von Willebrand factor (VWF) in blood fluid for the regulation of VWF-mediated platelet thrombus formation^1^. It has been reported that the functional deficiency of ADAMTS13 by autoantibodies or its gene mutation causes thrombotic thrombocytopenic purpura (TTP)^2^. In contrast, overactivation of ADAMTS13 by increased fluid shear stress causes excessive cleavage of large VWFs, resulting in VWF depletion and a bleeding disorder called acquired von Willebrand syndrome (aVWS)^3^. aVWS-like bleeding episodes have been frequently observed in patients with a mechanical circulatory assist device^4–6^, and attempts have been made to prevent and cure the bleeding disorder with mainly chemotherapy, surgery, and pump speed modulation^6^. However, these conventional treatment options have been frequently unsuccessful and raise the risks of thrombosis^7^. To address the medical issue for patients undergoing mechanical circulatory assist device support, a direct blockade of ADAMTS13-VWF interaction by monoclonal antibody (mAb) treatment is thought to be one of the therapeutic options^8^.

A mouse anti-ADAMTS13 antibody clone A10 (mA10) recognizes a disintegrin-like domain of ADAMTS13 and completely inhibited the VWF-cleaving activity of plasma ADAMTS13 in vitro^9^. For clinical application, the utilization of mouse mAbs has a limitation because the injection of mouse mAbs into a human body induces human anti-mouse antibody response (HAMA), resulting in the rapid clearance of the injected mAbs and the inability to bind and inhibit the target molecules^10–12^.

mAb humanization has played a key role for constructing human-like mAbs from non-human origins^13^. Currently, complementary determining region (CDR) grafting^14^ or its-related techniques^15^ have been widely used for mAb humanization. In this study, we constructed humanized A10 antibodies by CDR grafting with two human antibody frameworks, 8A7 and 16E8^16^, to develop an applicable therapeutic agent for the treatment of patients with ADAMTS13-related bleeding disorder. The characteristics of the two humanized A10 antibodies, namely A10/8A7 and A10/16E8, were assessed in vitro as well as in silico.

## Results

### Construction of humanized A10 antibodies A10/8A7 and A10/16E8 by CDR grafting

**Figure 1:**
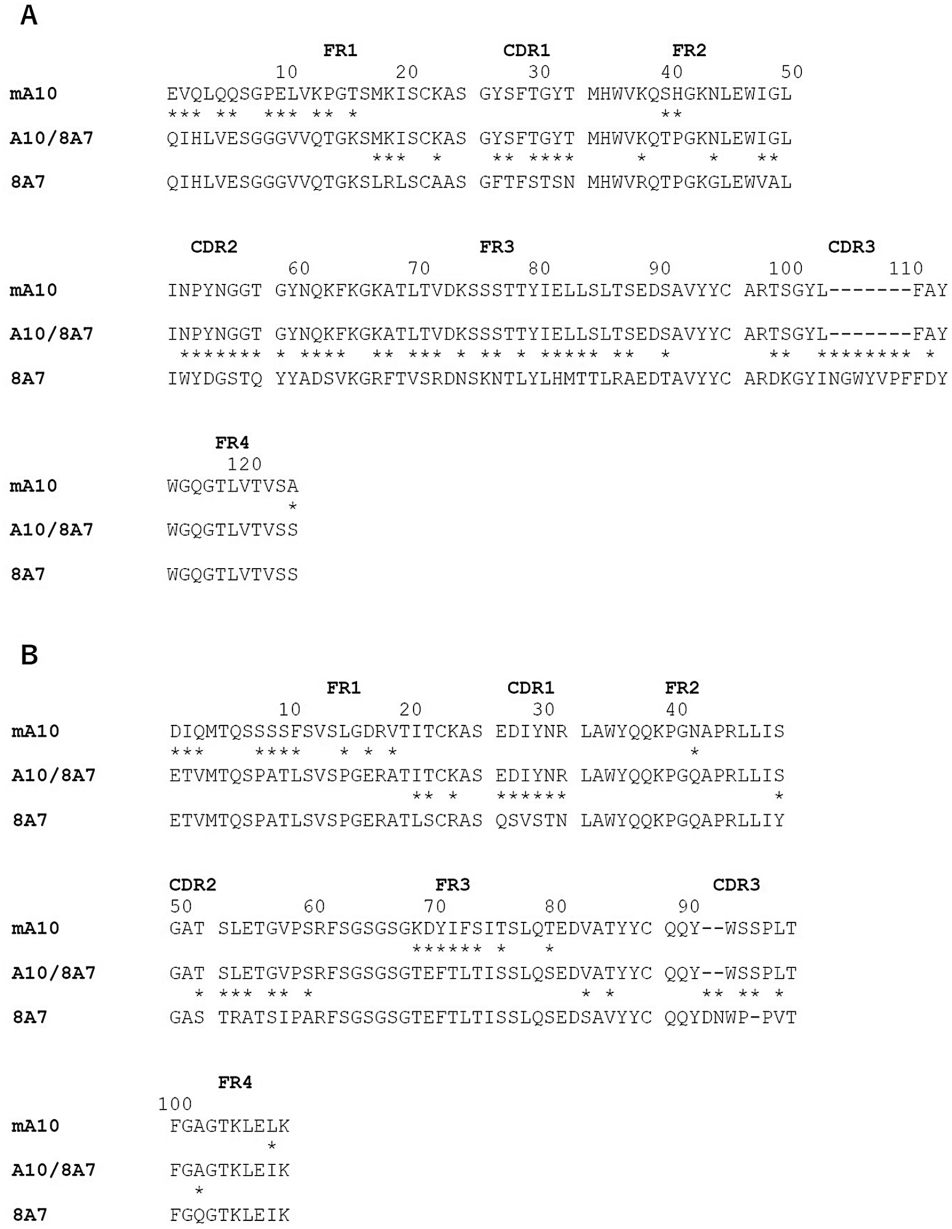
Amino acid sequence alignment of the heavy (**A**) and light (**B**) chain variable domains of mouse A10 (mA10), humanized A10/8A7, and human antibody framework 8A7. The FRs and CDRs in the variable domains of the mouse or human antibody sequences were determined by IMGT numbering scheme, respectively. Asterisks (*) indicate the difference between each paired amino acid sequence, and hyphens (−) indicate gaps.

**Figure 2:**
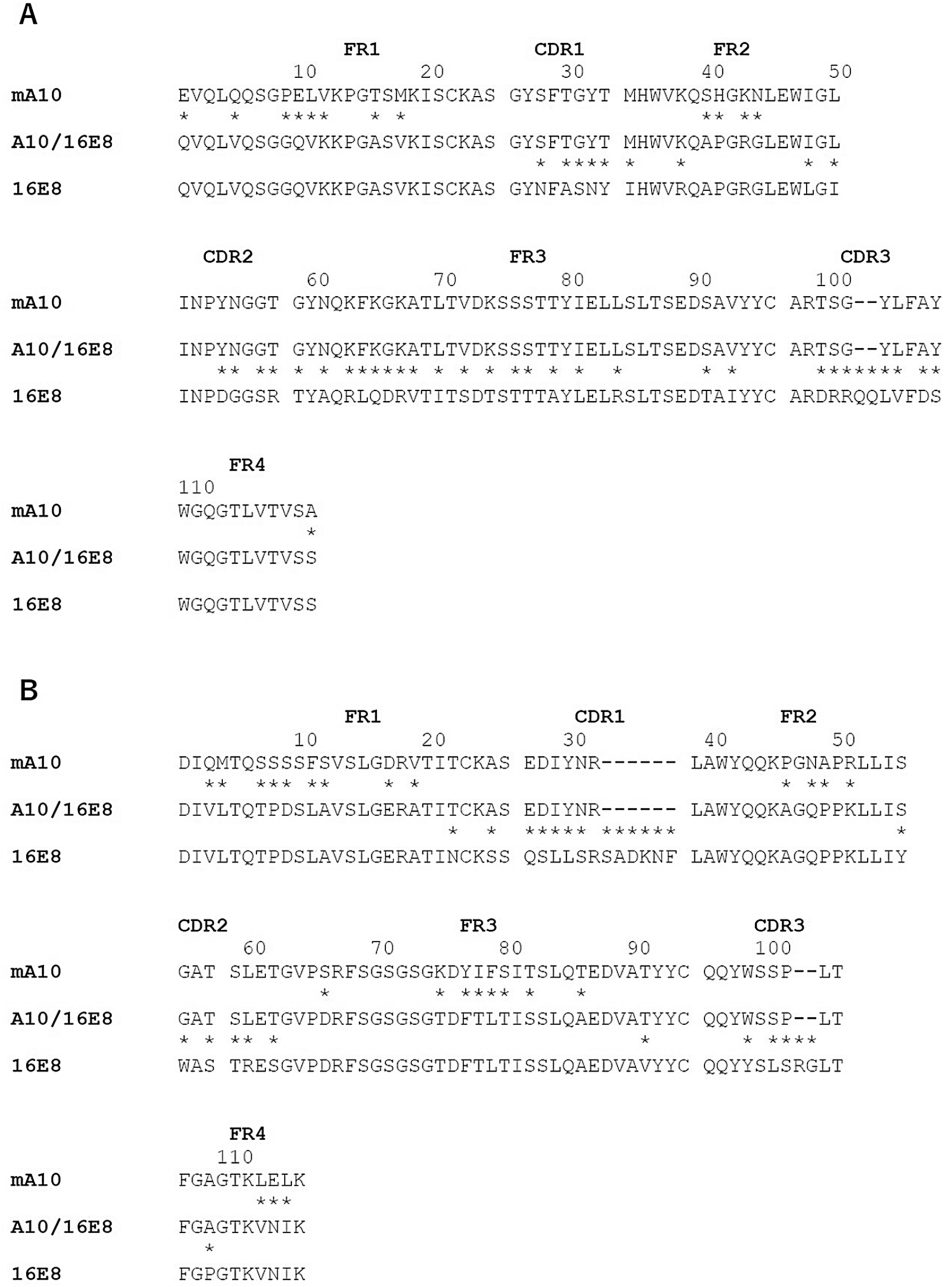
Amino acid sequence alignment of the heavy (**A**) and light (**B**) chain variable domains of mouse A10 (mA10), humanized A10/16E8, and human antibody framework 16E8. The FRs and CDRs in the variable domains of mouse or human antibody sequences were determined by IMGT numbering scheme, respectively. Asterisks (*) indicate the difference between each paired amino acid sequence, and hyphens (−) indicate gaps.

To design humanized A10 antibodies, the amino acid sequences of the heavy and light chain variable domains (V_H_ and V_L_, respectively) of mouse anti-ADAMTS13 antibody mA10 were compared with those of previously constructed human antibody frameworks, 8A7 and 16E8^16^. After determining the CDRs and framework regions (FRs) in the V_H_ and V_L_ of mA10, 8A7, and 16E8 in accordance with IMGT (the international ImMunoGeneTics information system) numbering scheme^17^, a global alignment was manually conducted by adjusting the positions and sequence motifs around CDRs between the amino acid sequences (Figs. 1,2). All CDRs and the flanking FR amino acid residues of mA10 were grafted into the corresponding regions of 8A7 and 16E8. The humanized A10 antibodies A10/8A7 and A10/16E8 contained a total of approximately 88% and 91% human content in the whole sequences, respectively (Figs. 1,2). Generally, humanized mAbs are constructed to be an average of 85% or more human content in the humanization process^18^. The humanized A10 antibodies constructed in this study have more than average number of human content in the whole amino acid sequences.

### Affinity determination of humanized A10 antibodies A10/8A7 and A10/16E8

**Figure 3:**
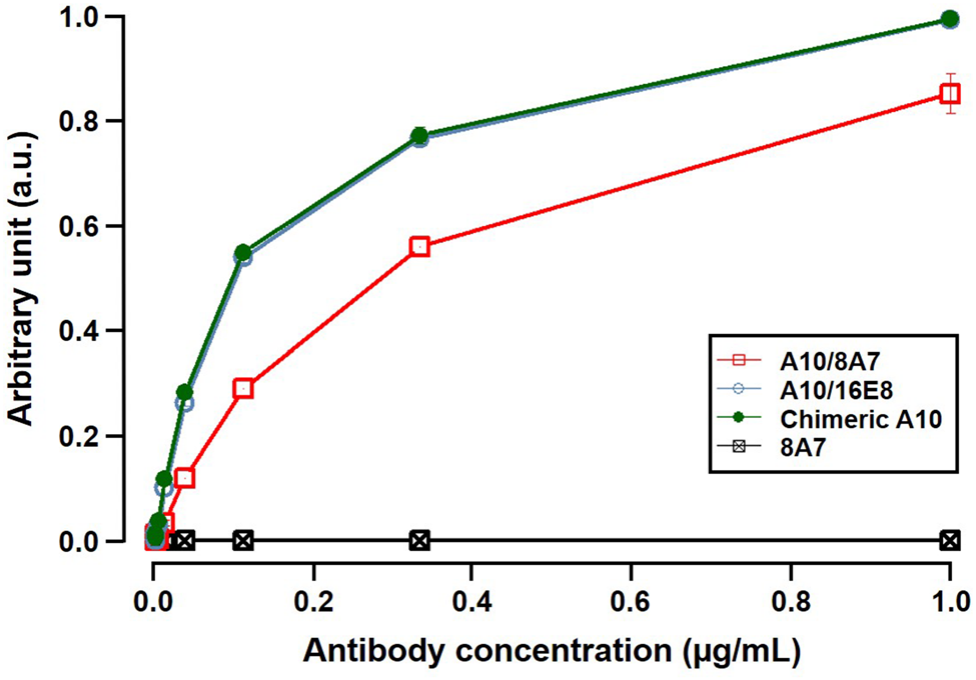
The dose–response relationship of A10/8A7 and A10/16E8 to ADAMTS13 in ELISA tests. Each plot in the dose–response curves was obtained in triplicate. Chimeric A10 and 8A7 were used as positive and negative controls, respectively. The horizontal axis is the concentration of antibodies used in the ELISA test. The vertical axis is standardized absorbance values (arbitrary unit) relative to the values of chimeric A10.

The recombinant humanized A10 antibodies A10/8A7 and A10/16E8 were transiently expressed in Expi293F cells, followed by antibody purification. The commercially available recombinant human ADAMTS13 protein was used for enzyme-linked immunosorbent assay (ELISA) and surface plasmon resonance (SPR) tests, to determine the antigen-binding affinity of A10/8A7 and A10/16E8 to ADAMTS13. The recombinant mA10 and human-mouse chimeric A10 (chimeric A10) were also generated and used to compare the binding affinity to the humanized A10 antibodies. As shown in Fig. 3, the binding affinity of A10/8A7 to ADAMTS13 was lower than that of chimeric A10. On the other hand, A10/16E8 binds to ADAMTS13 equally well compared with chimeric A10 (Fig. 3). The kinetic parameters of the purified mA10, chimeric A10, A10/8A7, and A10/16E8 were determined by SPR analysis. As a result, mA10, chimeric A10, A10/8A7, and A10/16E8 had slow dissociation rate. The dissociation constant (KD) value of A10/16E8 was not substantially different to that of mA10 and chimeric A10, while A10/8A7 showed the lower binding affinity, compared with other antibodies (Table 1). These results indicated that A10/16E8 successfully retains the binding affinity of the parental mA10 to ADAMTS13.

**Table 1:** Kinetic parameters of mA10, chimeric A10, A10/8A7, and A10/16E8 obtained by SPR analysis.

Antibody	*k*_a_^a^ (1/Ms)	*k*_d_^b^ (1/s)	*K*_D_^c^ (nM)
mA10	6.04 × 10^4^	3.62 × 10^–4^	5.99
Chimeric A10	7.27 × 10^3^	2.03 × 10^–5^	2.80
A10/8A7	9.27 × 10^3^	1.93 × 10^–4^	20.8
A10/16E8	1.24 × 10^4^	6.30 × 10^–5^	5.07

^a^Abbreviation: *k*_a_, association rate constant.

^b^Abbreviation: *k*_d_, dissociation rate constant.

^c^Abbreviation: *K*_D_, dissociation constant.

### Inhibition of ADAMTS13 activity by humanized A10 antibodies A10/8A7 and A10/16E8

**Figure 4:**
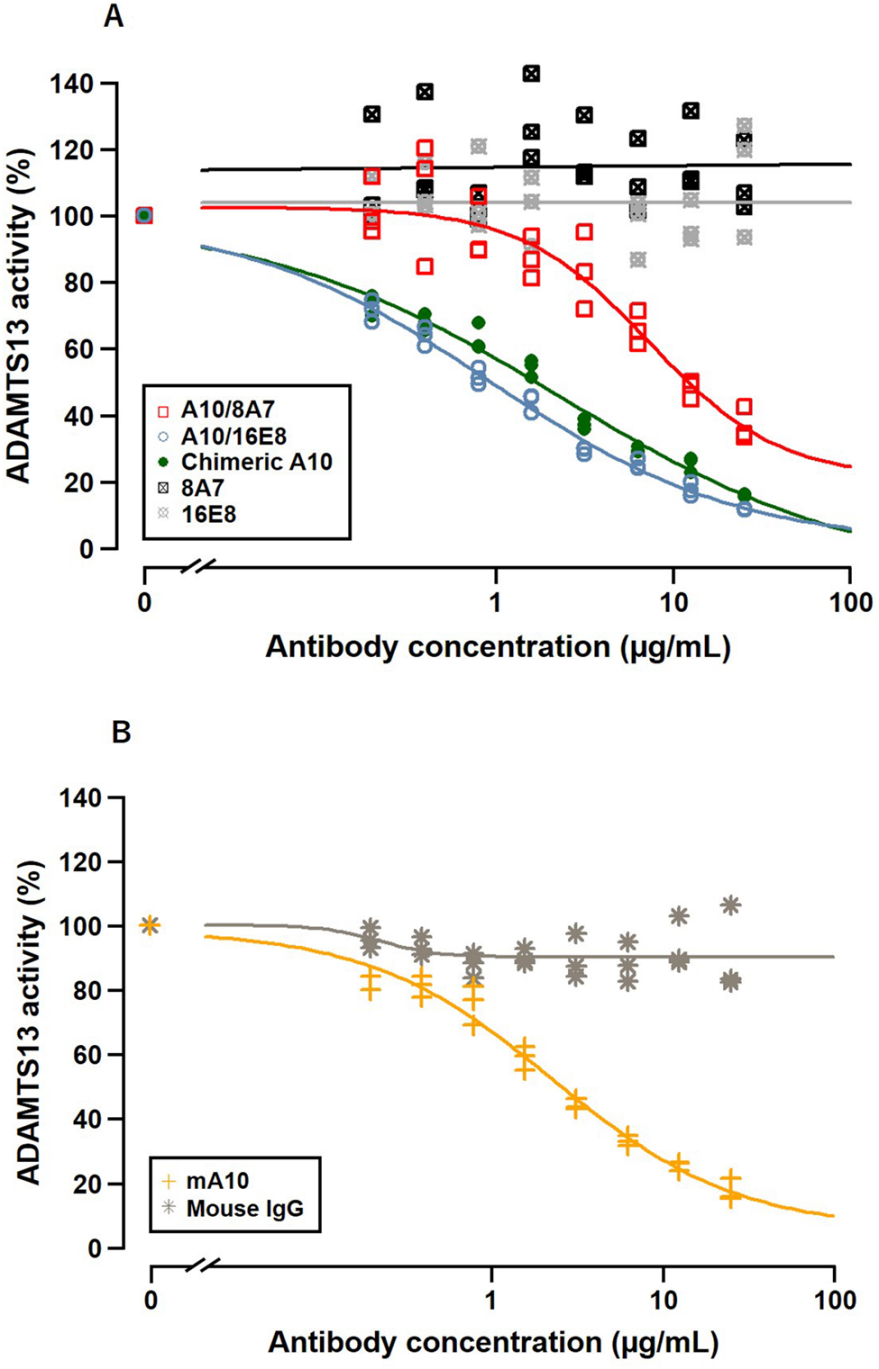
Monoclonal antibody concentration–response curves in vitro. The concentration-dependent ADAMTS13 inhibitory activity of human (**A**) and mouse (**B**) antibodies was determined by incubating normal pooled plasma with 2-fold serial dilutions of the antibodies ranging from 25 µg/mL to 0.20 µg/mL in triplicate. The values of dose 0 control were also measured to calculate the residual ADAMTS13 activity of each antibody. The horizontal axis is the concentration of antibodies used in these tests. The vertical axis is the residual plasma ADAMTS13 activity relative to the values of dose 0 control.

We evaluated the ADAMTS13 inhibitory activity of mA10, chimeric A10, A10/8A7, and A10/16E8 using a VWF-captured ELISA plate and compared each result. As shown in Fig. 4 and Table 2, A10/16E8 largely inhibited ADAMTS13 activity on VWF cleavage among the antibodies, while A10/8A7 showed the lower inhibitory activity compared with chimeric A10 and mA10. The obtained results in vitro indicated that A10/16E8 was successfully constructed as a humanized anti-ADATMS13 antibody with no loss of binding affinity and inhibitory activity.

**Table 2:** The half maximal inhibitory concentration (IC_50_) of mA10, chimeric A10, A10/8A7, and A10/16E8 obtained by ADAMTS13 inhibition assay.

Antibody	IC_50_^a^ (µg/mL)
mA10	2.28
Chimeric A10	2.30
A10/8A7	7.30
A10/16E8	0.90

^a^The IC_50_ values were estimated by using R version 3.6.2 packages.

### Antibody structure modeling and structural similarity analysis for mA10 and humanized A10 antibodies A10/8A7 and A10/16E8

**Figure 5:**
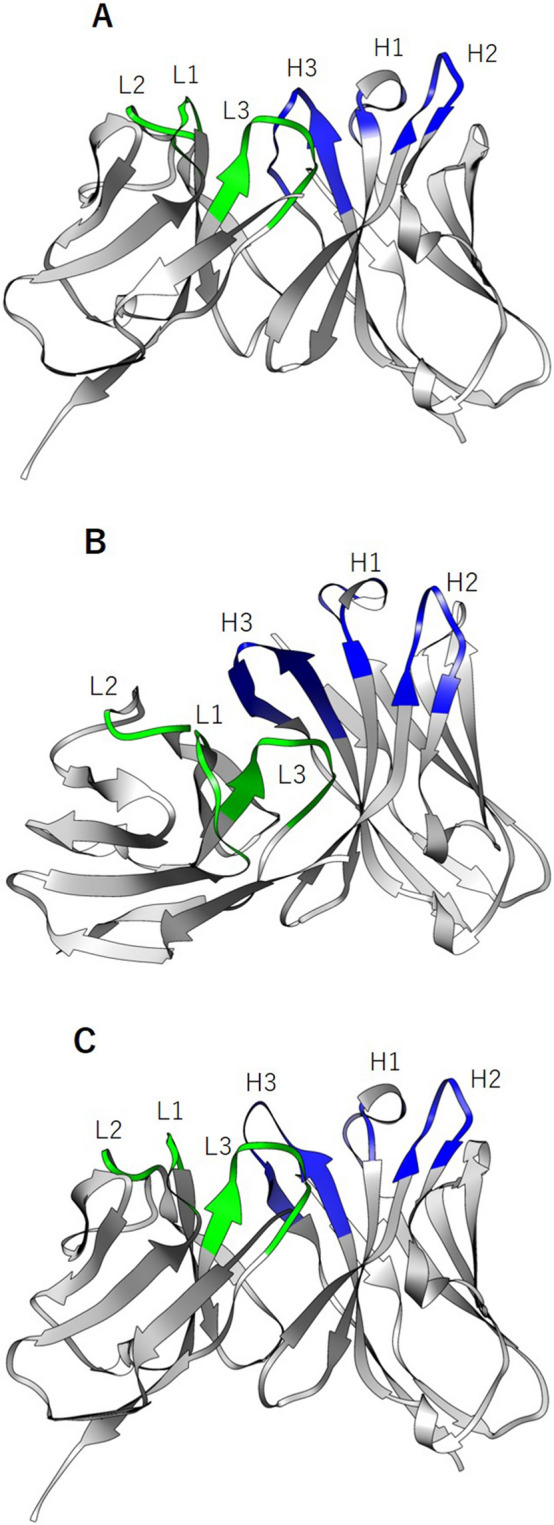
Predicted V_H_ and V_L_ structures of mA10 (**A**), A10/8A7 (**B**), and A10/16E8 (C) by the Rosetta software. The CDR loops of the heavy (H1–H3) and light (L1–L3) chains are indicated with blue and green, respectively.

To evaluate the structure similarity between mA10 and each of A10/8A7 and A10/16E8, we performed in silico antibody structure modeling by using Rosetta software^19^. The V_H_ and V_L_ structure models of mA10, A10/8A7, and A10/16E8 were generated by 1,000 runs of the structure modeling (Fig. 5), and the top 10 results for each antibody were used to calculate the values of mean root mean square deviation (RMSD) and Q-score (Supplemental Table S1). Here, both RMSD and Q-score are the measure of structural similarity between two protein structures, and the Q-score is the normalized RMSD values by alignment length to fairly compare protein structures of unequal length^20^. The smaller values of RMSD indicate higher structural similarity, whereas Q-score values range from 0 for completely dissimilar structures to 1 for identical structures^20^. Prior to the structural similarity analysis between mA10 and each of A10/8A7 and A10/16E8, we evaluated the fluctuation of the modeling results within each of antibody structure models by superimposing the top 10 structure models of each antibody. The Q-score values of V_H_ and V_L_ structure models were 1 and 1 for mA10, 0.96 and 1 for A10/8A7, and 0.95 and 1 for A10/16E8, respectively (Supplemental Table S1). These results indicated that the robustness of structure models within each of antibody structure models is high.

The structural similarity between mA10 and each of A10/8A7 and A10/16E8 was then evaluated using the structure models of these antibody. As a result, the mean RMSD values of both mA10-A10/8A7 and mA10-A10/16E8 pairs were less than 1 and the Q-score values of those were nearly 1 (Table 3), suggesting that the V_H_ and V_L_ structures of A10/8A7 and A10/16E8 are structurally similar to those of mA10. Among these results, relatively higher structural similarity was shown between the V_L_ structure models of mA10 and A10/16E8 according to the Q-score values (Table 3). These results obtained by our computational analyses suggest that A10/16E8 successfully retained a native form of mA10, resulting in the preservation of the binding affinity and inhibitory activity to ADAMTS13.

**Table 3:** Mean RMSD and Q-score for structural superposition between mA10 and each of two humanized A10 antibodies A10/8A7 and A10/16E8.

Antibody	RMSD^a,b^ (Å)		Q-score^a^	
	Heavy	Light	Heavy	Light
A10/8A7	0.48 ± 0.11	0.84 ± 0.01	0.96 ± 0.02	0.92 ± 0.00
A10/16E8	0.59 ± 0.13	0.71 ± 0.01	0.95 ± 0.02	0.95 ± 0.00

^a^The values of RMSD and Q-score were calculated using UCSF Chimera by superimposing the 100 pairs of the top 10 predicted structures of mA10 and each humanized A10 antibody.

^b^Abbreviation: RMSD, root mean square deviation.

## Discussion

Since the conventional treatment options for ADAMTS13-related bleeding disorder have not been always feasible and successful in patients receiving mechanical circulatory assist device support, we attempted to develop a new therapeutic agent by constructing humanized anti-ADAMTS13 antibodies in this study. The CDRs and the flanking FR amino acid residues of mouse anti-ADAMTS13 antibody A10 were grafted into the corresponding regions of human antibody frameworks 8A7 and 16E8. The humanized A10 antibody A10/16E8 retained the binding affinity and inhibitory activity of the parental mA10 to ADAMTS13, while the binding affinity and inhibitory activity of A10/8A7 were decreased compared with those of mA10. The computational antibody structure analysis was also performed and revealed that A10/16E8 successfully retained an original antibody structure of mA10.

To reduce the immunogenicity of mA10 for future clinical use, humanized A10 antibodies A10/8A7 and A10/16E8 were constructed, and their binding affinity and inhibitory activity to ADAMTS13 were evaluated in vitro. The binding affinity and inhibitory activity of A10/8A7 to ADAMTS13 were lower than those of mA10 and chimeric A10, while A10/16A8 showed the comparable binding activity and inhibitory activity to mA10 and chimeric A10 (Figs. 3,4, and Tables 1 and 2). These results indicated that A10/16E8 retained the binding affinity and inhibitory activity after CDR grafting. Previously published studies have attempted to improve CDR grafting techniques. Zhang and Ho tested several antibody numbering schemes to compare the different patterns of CDR loops on the structure of rabbit anti-mesothelin mAbs and determined several key amino acid residues necessary for the reservation of the original CDR loop conformation after CDR grafting^21^. Zhang and Ho also tested several human germline framework sequences to find a suitable human framework for CDR grafting and successfully constructed humanized anti-mesothelin mAbs from the parental rabbit antibodies with no binding affinity loss^21^. Margreitter et al. generated several variants of humanized mouse anti-idiotypic antibody Ab2/3H6 by substituting the amino acid residues in the FRs and identified the important sites responsible for the antigen binding^22^. Although insufficient experimental data and scientific evidence are available for optimizing CDR grafting techniques, a few key amino acid residues responsible for antigen binding affinity maturation are present within or around the CDRs of antibodies. In addition, selecting an appropriate human framework sequence for CDR grafting is an important step for successful humanization of non-human mAbs of interest. In this study, we cannot yet conclude which the amino acid residues around the CDRs of mA10 had a key role for the successful humanization of A10/16E8. On the other hand, the human antibody framework 16E8 was used as a successful framework to humanize mA10.

In this study, we employed computational methodologies to predict and compare the antibody structure of A10/8A7 and A10/16E8 to that of the parental mA10 to explain why the binding affinity and inhibitory activity of each antibody are different. The values of RMSD and Q-score were calculated to quantitatively evaluate the structural similarity of the structure models. The generally acceptable criterion of RMSD for protein structure comparison is 2 Å^23^. The mean RMSD values of mA10-A10/8A7 and mA10-A10/16E8 pairs were below the criterion (Table 3), suggesting that the structure of both A10/8A7 and A10/16E8 is similar to that of mA10. According to the Q-score values, no substantial difference was found between mA10-A10/8A7 and mA10-A10/16E8 pairs in terms of the V_H_ structure similarity, whereas the mA10-A10/16E8 pair showed slightly higher similarity of V_L_ structure, compared with the mA10-A10/8A7 pair. These results are consistent with the visible difference of the antibody structure models between mA10 and each of A10/8A7 and A10/16E8. The V_L_ structure of A10/8A7 seems dissimilar to that of mA10 and A10/16E8, while both the V_H_ and V_L_ structures of A10/16E8 seem nearly identical to those of mA10 (Fig. 5). These observations suggest that the antibody structure of A10/8A7 is apart from the native form of the parental ADAMT13-binding antibody, while A10/16E8 could retain the native form. A previously published study has reported that retaining the original antibody structure in an antibody humanization process may enable the humanized antibodies to also retain the binding affinity of the original antibodies^24^. In addition, Foote and Winter have reported that essential amino acid residues are present in FRs for the successful CDR loop transplants of original antibodies in CDR grafting^25^. Less is known about how and to what extent the difference in the antibody structure affects the resulting antigen binding affinity and inhibitory activity. However, these observations in our computational analyses are possible reasons to explain why the humanized A10 antibodies constructed in this study could or could not retain the binding affinity and inhibitory activity of mA10 to ADAMTS13.

In conclusion, our results demonstrated that we successfully constructed humanized A10 antibodies in this study. The humanized A10 antibody A10/16E8 retained the binding affinity and inhibitory activity of mA10 to ADAMTS13 after CDR grafting and is expected to have lower immunogenicity in humans for clinical application. A10/16E8 has potential application in the treatment of ADAMTS13-related bleeding disorder in patients undergoing mechanical circulatory assist device support.

## Methods

### Human antibody frameworks 8A7 and 16E8

Previously constructed human antibody clones, 8A7 and 16E8^16^, were used as human antibody frameworks and negative controls in this study.

## Construction of mA10 and chimeric A10 antibody expression vectors

The total RNA of mA10 hybridoma cells was extracted and purified using TRIzol™ Reagent (Thermo Fisher Scientific, 15596018) and RNeasy Mini Kit (QIAGEN, 74106). The extracted RNA was used for nested reverse transcription-polymerase chain reaction (RT-PCR) using SMART cDNA Library Construction Kit (Takara, 634901) according to the manufacturer's instructions. PCR primers used in this study are listed in Supplemental Table S2. In the RT-PCR procedure, *Not*I and *Eco*RI sites were added to the 5′ and 3′ end of the mA10 genes, respectively. The PCR products were then digested with *Not*I and *Eco*RI, and the heavy and light chain genes of mA10 were inserted into pQEFIP or pQEFIN vectors^16^, respectively.

To construct the expression vectors of chimeric A10 antibody, the V_H_ and V_L_ genes of mA10 were combined with the heavy and light chain constant domain (C_H_, and C_L_, respectively) genes of 8A7, respectively. The constructed mA10 plasmids were used to amply the V_H_ and V_L_ genes of mA10. Previously constructed 8A7 plasmids^16^ were used to amplify the C_H_ and C_L_ genes because 8A7 has typical sequences of C_H_ and C_L_ for immunoglobulin gamma 1 (IgG1) and immunoglobulin kappa (Igκ), respectively. Using a PCR-based method with target specific primers (Supplemental Table S2), the expression vectors were also amplified along with the C_H_ and C_L_ genes for cloning, and approximately 20-nt overlaps were added to the both 5′and 3′ end of the target genes, respectively. The PCR products were then enzymatically assembled using NEBuilder HiFi DNA Assembly Master Mix (NEB, E2621L), in order to combine V_H_ and V_L_ genes of mA10 with the C_H_ and C_L_ genes of 8A7, respectively.

### Construction of humanized A10 antibody expression vectors

The gene sequences of mA10, 8A7, and 16E8 were used for an NCBI IgBLAST (http://www.ncbi.nlm.nih.gov/igblast/) search against IMGT database^17^ with default settings, in order to determine the CDRs and FRs in V_H_ and V_L_ genes of mA10, 8A7, and 16E8 (Figs. 1,2). The genes of humanized A10/8A7 and A10/16E8 antibodies were constructed by grafting the CDRs and the flanking FRs of mA10 gene onto the corresponding regions of 8A7 and 16E8 genes, respectively. The V_H_ and V_L_ genes of A10/8A7 and A10/16E8 were synthesized by Eurofins Genomics (Tokyo, Japan) following codon optimization. Previously constructed plasmids of 8A7 and 16E8 genes were used to amplify the C_H_ and C_L_ genes along with the expression vectors^16^. Using a PCR-based method (Supplemental Table S2), approximately 20-nt overlaps were added to the both 5’ end and 3’ end of the target genes, respectively, followed by the assembly of the PCR products described above.

### Transient expression and purification of mA10, chimeric A10, and humanized A10 antibodies

The appropriate pairs of heavy and light chain expression vectors for producing mA10, chimeric A10, A10/8A7, and A10/16E8 mAbs were co-transfected into Expi293F cells using ExpiFectamine™ 293 Transfection Kit (Thermo Fisher Scientific, A14524) according to the manufacturer’s instructions. After 5 days of cell culture, the culture supernatant was collected and centrifuged twice at 2380 × *g* for 5 min at room temperature and subsequently at 8000 × *g* for 10 min at 4℃. The centrifugation supernatant was filtered using a 0.45 µm disc filter (Millipore, SLHV033RS), and the antibodies contained in the flow-through fraction were purified by protein G affinity chromatography using a 1 mL HiTrap Protein GHP column (Cytiva, 17,040,401) as previously described^16^. The purity of the purified antibodies was checked by sodium dodecyl sulfate polyacrylamide gel electrophoresis (SDS-PAGE) (Supplemental Fig. S1). The purified antibody concentration was measured using NanoDrop One (Thermo Fisher Scientific) at a wavelength of 280 nm.

### ELISA

The antigen binding affinity and specificity of the purified A10/8A7 and A10/16E8 mAbs were evaluated by ELISA. Briefly, flat-bottom 96-well microtiter plates (Thermo Fisher Scientific, 167,008) were coated with Recombinant Human ADAMTS13 (Full Length) Protein (R&D Systems, 6156-AD-020) at 50 ng/well, incubated overnight at 4℃, and blocked with ELISA blocking buffer (1 × phosphate-buffered saline (PBS), 2% bovine serum albumin (BSA), 0.05% NaN_3_) at room temperature for 1 h. Three-fold serial dilutions of the mAbs from 1 µg/mL to 0.001 µg/mL in ELISA diluent (1 × PBS, 1% BSA, 0.05% NaN_3_) were incubated in triplicate on the protein-coated wells for 1 ~ 2 h. Chimeric A10 and mA10 were used as positive controls, and 8A7 and 16E8, and mouse IgG2b-UNLB (SouthernBiotech, 1090–01) were used as negative controls for human and mouse ELISA tests, respectively. After washing the ELISA plates, human or mouse mAbs bound to the coated ADAMTS13 protein were detected by Goat Anti-Human IgG-AP (SouthernBiotech, 2040–04) or Goat Anti-Mouse IgG-AP (SouthernBiotech, 1030–04) with an alkaline phosphatase substrate (Sigma-Aldrich, S0942), respectively. The absorbance at 405 nm (A_405_) and 650 nm (A_650_) was read using a Multimode Plate Reader Enspire instrument (PerkinElmer), and A_650_ was subtracted from A_405_. The ELISA results for 16E8, mA10, and mouse IgG2b are shown as supplementary data (Supplemental Fig. S2).

### SPR

To determine the kinetic parameters of the purified A10/8A7 and A10/16E8 mAbs, such as KD, SPR analyses were carried out using a Biacore T200 instrument (Cytiva). According to the manufacturer’s instructions, anti-human and anti-mouse IgG (Fc) antibodies were immobilized onto Series S Sensor Chip CM5 (Cytiva, 29,104,988) using Human Antibody Capture Kit (Cytiva, BR-1008–39) and Mouse Antibody Capture Kit (Cytiva, BR-1008–38), respectively, with Amine Coupling Kit (Cytiva, BR100050). All the antibodies analyzed were captured at approximately 1,000 response unit (RU). Chimeric A10 and mA10 were used as positive controls, and 8A7 and 16E8, and mouse IgG2b-UNLB (SouthernBiotech, 1090–01) were used as negative controls for SPR analyses of human and mouse antibodies, respectively. The binding curves were obtained by injecting 2-fold serial dilutions of Recombinant Human ADAMTS13 (Full Length) Protein (R&D Systems, 6156-AD-020) ranging from 80 nM to 0.625 nM in PBS containing 0.05% Tween 20 (PBS-T) (Supplemental Fig. S3 and S4). The operation parameters were as follows: temperature, 25 ℃; flow rate, 30 μL/min; contact time, 240 s; dissociation time, 900 s. The obtained binding curves of each antibody were analyzed using 1:1 binding model in Biacore T200 Evaluation Software version 2.0 (Cytiva).

### ADAMTS13 inhibition assay

ADAMTS13 activity was measured by chromogenic ELISA (Kainos Inc., CY-6000)^26^ in triplicate. For inhibition assays, residual ADAMTS13 activity was measured after incubating normal pooled plasma with 2-fold serial dilutions of the antibodies ranging from 25 µg/mL to 0.20 µg/mL. Chimeric A10 and mA10 were used as positive controls, and 8A7 and 16E8, and mouse IgG (FUJIFILM Wako Pure Chemical Co., 140–09,511) were used as negative controls for ADAMTS13 inhibition assays of human and mouse antibodies, respectively. The concentration–response relationship between antibody concentration and residual ADAMTS13 activity was fitted to a sigmoid model using R version 3.6.2 packages.

### Antibody structure modeling and structural similarity analysis

The three-dimensional (3-D) structure of mA10, A10/8A7, and A10/16E8 was predicted using the locally installed Rosetta software (version 2020.37)^19^. Briefly, amino acid sequences of V_H_ and V_L_ of the antibodies were used for a BLASTp search against the PDB database, in order to generate antibody structure modeling templates. One thousand runs of antibody structure modeling were performed using the RosettaAntibody software with the generated templates, and the top 10 antibody structure models were selected according to the modeling results. The selected antibody structure models were visualized and analyzed using the UCSF Chimera^27^. The values of RMSD and Q-score were calculated to evaluate the structural similarity between mA10 and each of A10/8A7 and A10/16E8, where RMSD is the measure of the average distance between the Cα atoms of superimposed protein structures and Q-score is the structural similarity score ranging from 0 to 1; Q-score is 1 for completely identical or superimposed structures and decreases as the similarity decreases^20^. The fluctuation of the modeling results within each of antibody structure models was also evaluated by superimposing the top 10 antibody structure models of each antibody (Supplemental Table S1).

### Data availability

All the data are supplied within this article, and the full datasets can be provided upon request.

## Supplementary Information

Supplementary Information.
