# Supplementary material for: Optimization of anti-ADAMTS13 antibodies for the treatment of ADAMTS13-related bleeding disorder in patients receiving circulatory assist device support
Source: Sci Rep. 2021 Nov 16;11:22341. doi: 10.1038/s41598-021-01696-3 (PMC8595387; doi:10.1038/s41598-021-01696-3)
Supplement: Supplementary file 1 — Supplementary Information. [file 41598_2021_1696_MOESM1_ESM.pdf]

Scientific Reports

Title

Optimization of anti-ADAMTS13 antibodies for the treatment of ADAMTS13-related bleeding disorder in patients receiving circulatory assist device support

Authors

Toshihiro Ito[1,†], Takeharu Minamitani[2,3,†,††], Masaki Hayakawa[4], Ryota Otsubo[2,3], Hiroki Akiba[5,6], Kouhei Tsumoto[7,8,9], Masanori Matsumoto[4*], Teruhito Yasui[2,3,10*]

Affiliations

[1] Laboratory of Proteome Research, National Institutes of Biomedical Innovation, Health and Nutrition (NIBIOHN), 7-6-8 Saito-Asagi, Ibaraki City, Osaka, 567-0085, Japan

[2] Laboratory of Infectious Diseases and Immunity, National Institutes of Biomedical Innovation, Health and Nutrition (NIBIOHN), 7-6-8 Saito-Asagi, Ibaraki City, Osaka, 567-0085, Japan

[3] Laboratory of Immunobiologics Evaluation, Center for Vaccine and Adjuvant Research (CVAR), National Institutes of Biomedical Innovation, Health and Nutrition (NIBIOHN), 7-6-8 Saito-Asagi, Ibaraki City, Osaka, 567-0085, Japan

[4] Department of Blood Transfusion Medicine, Nara Medical University, 840 Shijo-cho, Kashihara City, Nara, 634-8522, Japan

[5] Laboratory of Advanced Biopharmaceuticals, Center for Drug Design Research (CDDR), National Institutes of Biomedical Innovation, Health and Nutrition

(NIBIOHN), 7-6-8 Saito-Asagi, Ibaraki City, Osaka, 567-0085, Japan

[6] Graduate School of Pharmaceutical Sciences, Kyoto University, 46-29 Yoshida-shimoadachicho, Sakyo-ku, Kyoto 606-8501, Japan

[7] Center for Drug Design Research (CDDR), National Institutes of Biomedical Innovation, Health and Nutrition (NIBIOHN), 7-6-8 Saito-Asagi, Ibaraki City, Osaka, 567-0085, Japan

[8] Medical Proteomics Laboratory, The Institute of Medical Science, The University of Tokyo, 4-6-1 Shirokanedai, Minato-ku, Tokyo 108-8639, Japan

[9] Department of Bioengineering, School of Engineering, The University of Tokyo, 7-3-1 Hongo, Bunkyo-Ku, Tokyo 113-8656, Japan

[10] Laboratory of Pharmaceutical Integrated Omics, Department of Pharmaceutical Engineering, Facility of Engineering, Toyama Prefectural University, 5180 Kurokawa, Imizu, Toyama 939-0398, Japan

† These authors contributed equally to this work.

†† Present address: Toyama Prefectural Institute for Pharmaceutical Research, 17-1 Nakataikoyama, Imizu-City, Toyama, 939-0363, Japan

Contact information

*Corresponding authors

Teruhito Yasui (communicates with the Editorial and Production offices)

Postal address: Laboratory of Infectious Diseases and Immunity, National Institutes of Biomedical Innovation, Health and Nutrition (NIBIOHN), 7-6-8 Saito-Asagi, Ibaraki City, Osaka, 567-0085, Japan
E-mail address: tyasui@nibiohn.go.jp
Telephone number: +81-72-641-9902
Fax number: +81-72-641-9903

Masanori Matsumoto
Postal address: Department of Blood Transfusion Medicine, Nara Medical University, 840 Shijo-cho, Kashihara City, Nara, 634-8522, Japan
E-mail address: mmatsumo@naramed-u.ac.jp
Telephone number: +81-744-22-3051
Fax number: +81-744-29-0771

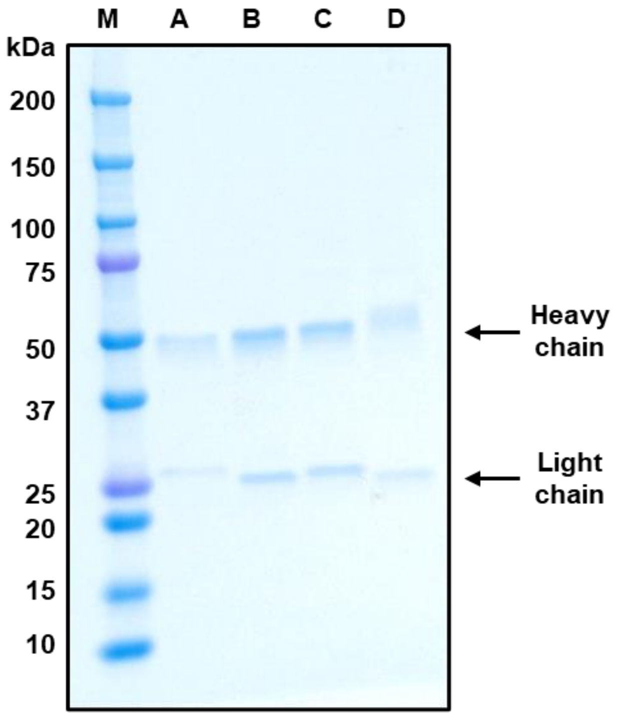

Supplemental Figure S1. Sodium dodecyl sulfate polyacrylamide gel electrophoresis (SDS-PAGE) analysis for the purity check of purified A10/8A7 (A), A10/16E8 (B), human-mouse chimeric A10 (C), and mouse A10 (mA10) (D) monoclonal antibodies in protein G affinity chromatography. The heavy and light chains of the antibodies appeared on the SDS-PAGE with approximately 50 kDa and 25 kDa, respectively.

A

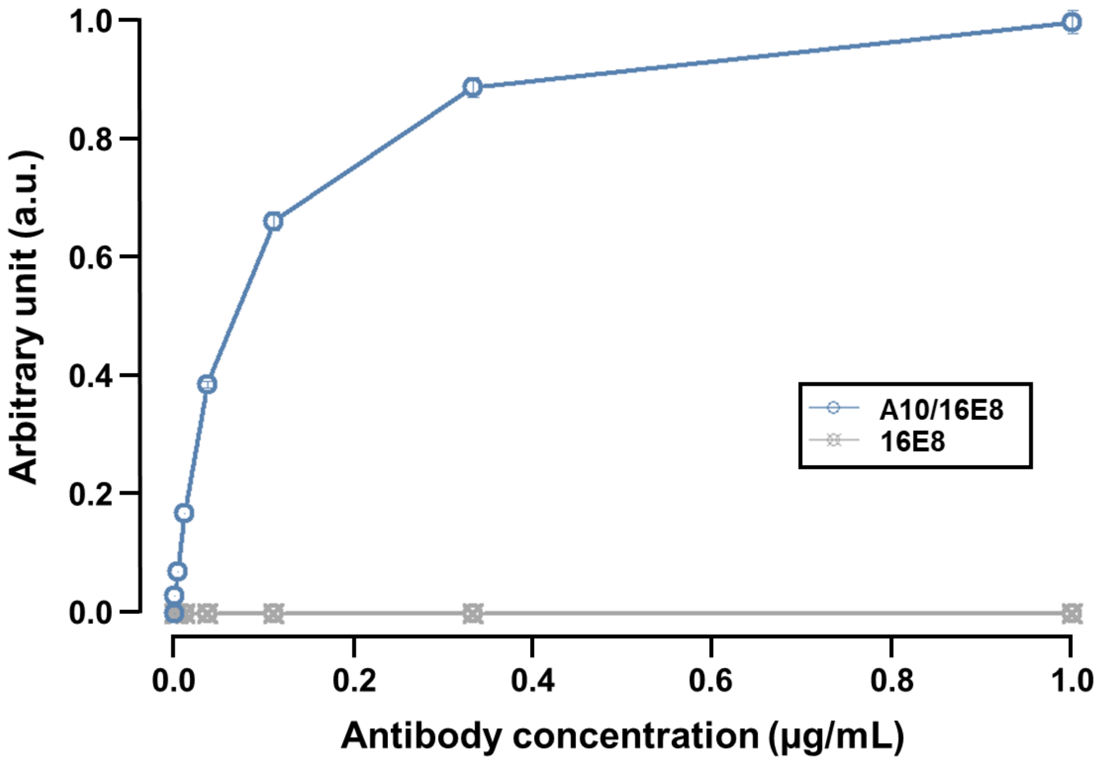
Arbitrary unit (a.u.)
1.0
0.8
0.6
0.4
0.2
0.0
0.0
0.2
0.4
0.6
0.8
1.0
Antibody concentration (μg/mL)
A10/16E8
16E8

B

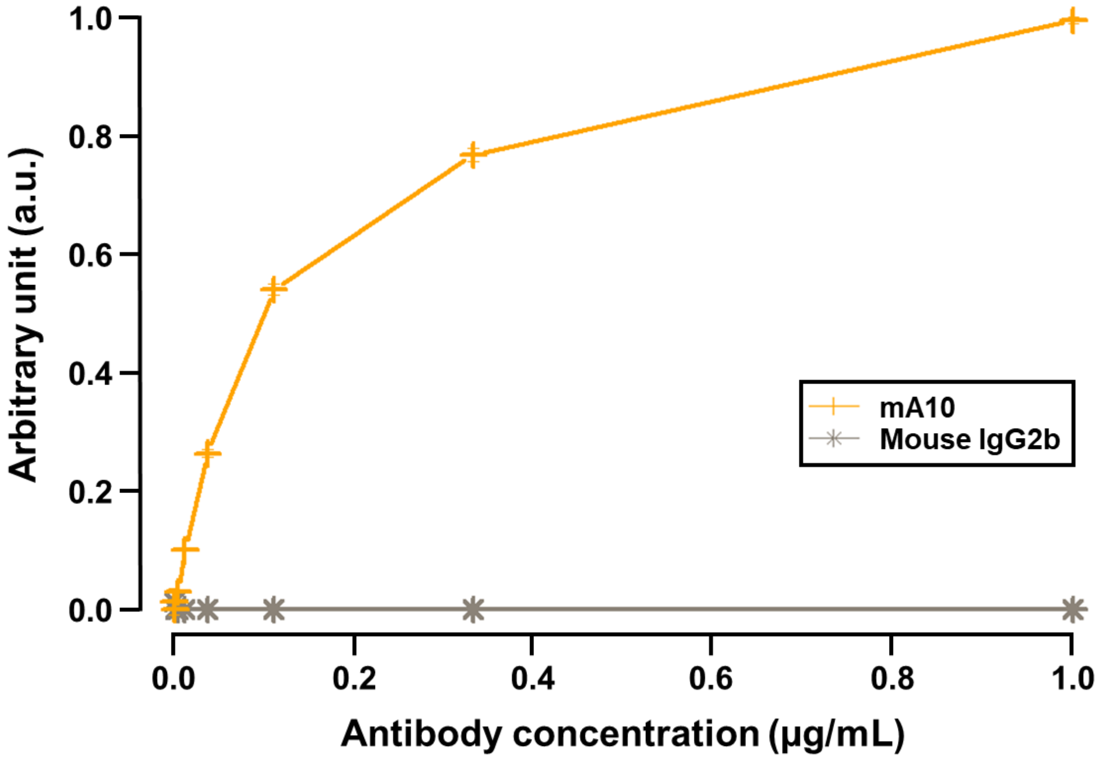
Arbitrary unit (a.u.)
1.0
0.8
0.6
0.4
0.2
0.0
0.0
0.2
0.4
0.6
0.8
1.0
Antibody concentration (μg/mL)
mA10
Mouse IgG2b

Supplemental Figure S2. The dose-response relationship of A10/16E8 (A) and mA10 (B) to ADAMTS13 (a disintegrin-like and metalloproteinase with thrombospondin type-1 motif 13) in enzyme-linked immunosorbent assay (ELISA) tests. Each plot in the dose-response curves was obtained in triplicate. 16E8 and mouse IgG2b were used as negative controls for human and mouse ELISA tests, respectively. The horizontal axis is the concentration of antibodies. The vertical axis is standardized absorbance values (arbitrary unit) relative to the values of A10/16E8 and mA10 in each ELISA test.

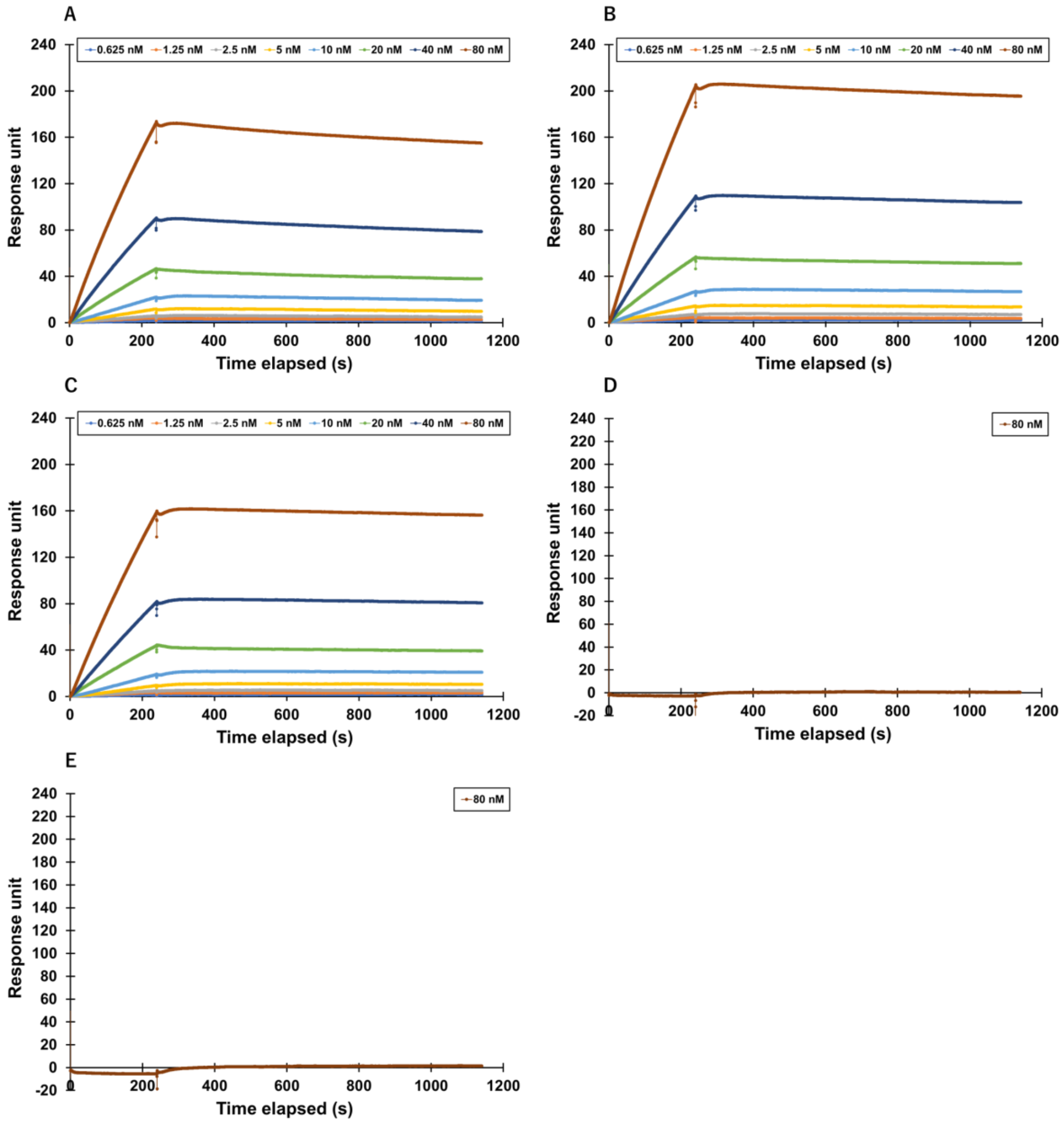

Supplemental Figure S3. Surface plasmon resonance (SPR) analyses for A10/8A7 (A), A10/16E8 (B), chimeric A10 (C), 8A7 (D), and 16E8 (E). The binding curves were obtained by injecting 2-fold serial dilutions of the ADAMTS13 protein ranging from 80 nM to 0.625 nM for chimeric A10, A10/8A7, and A10/16E8. 8A7 and 16E8 were used as negative controls for SPR analyses of human antibodies, and the binding curve was obtained by injecting 80 nM ADAMTS13. The contact time and dissociation time were set as 240 s and 900 s, respectively.

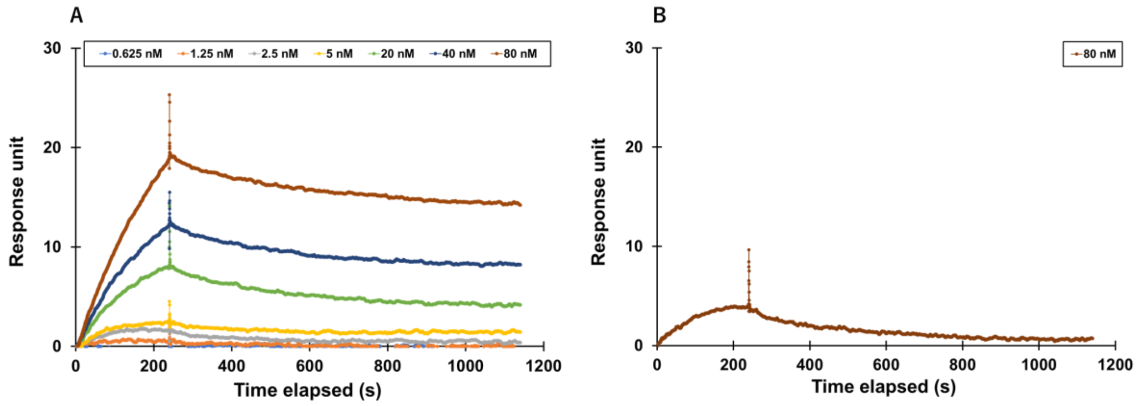

Supplemental Figure S4. SPR analyses for mA10 (A) and mouse IgG2b (B). The binding curves were obtained by injecting 2-fold serial dilutions of the ADAMTS13 protein ranging from 80 nM to 0.625 nM for mA10. Mouse IgG2b was used as a negative control for SPR analyses of mouse antibodies, and the binding curve was obtained by injecting 80 nM ADAMTS13. The contact time and dissociation time were set as 240 s and 900 s, respectively.

Supplemental Table S1. Root mean square deviation (RMSD) and Q-score[1] obtained by superimposing the 10 predicted structures of each antibody.

Antibody		RMSD[a] (Å)	Q-score[a]
mA10	Heavy	0.20	1.00
	Light	0.07	1.00
A10/8A7	Heavy	0.60	0.96
	Light	0.09	1.00
A10/16E8	Heavy	0.70	0.95
	Light	0.18	1.00

a. The values of RMSD and Q-score were calculated using the UCSF Chimera[2].

Supplemental Table S2. Polymerase chain reaction (PCR) primers used in this study

Antibody	Target gene	Chain		Sequence (5′–3′)
mA10	mA10 whole gene	1st PCR primers for heavy chain	Forward[a]	AAGCAGTGGTATCAACGCAGAGT
			Reverse	TAGGACCTGAGAGCTTTGTGGGTGCTGAGC
		1st PCR primers for light chain	Forward[a]	AAGCAGTGGTATCAACGCAGAGT
			Reverse	GCTGGTGGTGGCGTCTCAGGACCTTTGTCT
		2nd PCR primers for heavy chain	Forward[b]	GGGGCGGCCGCAGAGTGGCCATTACGGCCGGG
			Reverse	GGGGAATTCTCATTTACCCGGAGACCGGGAGATGG
		2nd PCR primers for light chain	Forward[b]	GGGGCGGCCGCAGAGTGGCCATTACGGCCGGG
			Reverse	GGGGAATTCCTAACACTCATTCCTGTTGAAGCTCTTG
Chimeric A10	mA10 variable domain	Heavy	Forward	GAATTGGTGACCGCGGCCGCAGAGTGGCCATTACGG
			Reverse	GGGCCCTTGGTGGAGGCTGCAGAGACAGTGACCAGA
		Light	Forward	GAATTGGTGACCGCGGCCGCAGAGTGGCCATTACGG
			Reverse	GATGGTGCAGCCACAGTTCGTTTCAGCTCCAGCTTGGTCCC
	8A7 constant domain	Heavy	Forward	GCCTCCACCAAGGGCCCATC
			Reverse	GCGGCCGCGGTCACCAATTC
		Light	Forward	CGAACTGTGGCTGCACCATC
			Reverse	GCGGCCGCGGTCACCAATTC

See next page.

Antibody	Target gene	Chain		Sequence (5′–3′)
Humanized antibody	A10/8A7 variable domain	Heavy	Forward	AATTGGTGACCGCGGCCGCCACCATGGAGTTCGGCCTGAG
			Reverse[c]	GATGGGCCCTTGGTGGAGGCGCTGCTCACGGTCACCAGGGTGC
		Light	Forward	GTGACCGCGGCCGCGTCGACACCATGGAGGCCCCCGCCCA
			Reverse	GATGGTGCAGCCACAGTTCGCTTGATCTCCAGCTTGGTGC
	A10/16E8 variable domain	Heavy	Forward	AATTGGTGACCGCGGCCGCCACCATGGACTGGACCTGGAG
			Reverse[c]	GATGGGCCCTTGGTGGAGGCGCTGCTCACGGTCACCAGGGTGC
		Light	Forward	GTGACCGCGGCCGCGTCGACACCATGGTGCTGCAGACCCA
			Reverse	GATGGTGCAGCCACAGTTCGCTTGATGTTCACCTTGGTGC
	8A7 and 16E8 constant domain	Heavy	Forward	GCCTCCACCAAGGGCCCATC
			Reverse	GGCGGCCGCGGTCACCAATT
		Light	Forward	CGAACTGTGGCTGCACCATC
			Reverse	GTCGACGCGGCCGCGGTCAC

a. The same forward PCR primer was used in the 1st PCR reaction for the amplification of heavy and light chain genes of mA10.

b. The same forward PCR primer was used in the 2nd PCR reaction for the amplification of heavy and light chain genes of mA10.

c. The same PCR primer sets were used for the amplification of heavy and light chain constant domain genes of A10/8A7 and A10/16E8, respectively.

References

1. Krissinel, E. & Henrick, K. Secondary-structure matching (SSM), a new tool for fast protein structure alignment in three dimensions. *Acta Crystallogr. Sect. D Biol. Crystallogr.* **60**, 2256–2268 (2004).
2. Pettersen, E. F. *et al.* UCSF Chimera - A visualization system for exploratory research and analysis. *J. Comput. Chem.* **25**, 1605–1612 (2004).
